# Interactive machine learning for health informatics: when do we need the human-in-the-loop?

**DOI:** 10.1007/s40708-016-0042-6

**Published:** 2016-03-02

**Authors:** Andreas Holzinger

**Affiliations:** 1 Research Unit, HCI-KDD, Institute for Medical Informatics, Statistics & Documentation, Medical University Graz, Graz, Austria; 2 Institute for Information Systems and Computer Media, Graz University of Technology, Graz, Austria

**Keywords:** Interactive machine learning, Health informatics

## Abstract

Machine learning (ML) is the fastest growing field in computer science, and health informatics is among the greatest challenges. The goal of ML is to develop algorithms which can learn and improve over time and can be used for predictions. Most ML researchers concentrate on automatic machine learning (aML), where great advances have been made, for example, in speech recognition, recommender systems, or autonomous vehicles. Automatic approaches greatly benefit from big data with many training sets. However, in the health domain, sometimes we are confronted with a small number of data sets or rare events, where aML-approaches suffer of insufficient training samples. Here interactive machine learning (iML) may be of help, having its roots in reinforcement learning, preference learning, and active learning. The term iML is not yet well used, so we define it as *“algorithms that can interact with agents and can optimize their learning behavior through these interactions, where the agents can also be human.”* This *“human-in-the-loop”* can be beneficial in solving computationally hard problems, e.g., subspace clustering, protein folding, or k-anonymization of health data, where human expertise can help to reduce an exponential search space through heuristic selection of samples. Therefore, what would otherwise be an NP-hard problem, reduces greatly in complexity through the input and the assistance of a human agent involved in the learning phase.

## Introduction

Originally the term “machine learning” was defined as *“... artificial generation of knowledge from experience,”* and the first studies have been performed with games, i.e., with the game of checkers [1].

Today, machine learning (ML) is the fastest growing technical field, at the intersection of informatics and statistics, tightly connected with data science and knowledge discovery, and health is among the greatest challenges [2, 3].

Particularly, probabilistic ML is extremely useful for health informatics, where most problems involve dealing with uncertainty. The theoretical basis for the probabilistic ML was laid by Thomas Bayes (1701–1761), [4, 5]. Probabilistic inference vastly influenced artificial intelligence and statistical learning and the inverse probability allows to infer unknowns, learn from data and make predictions [6, 7].

Recent progress in ML has been driven both by the development of new learning algorithms and theory and by the ongoing explosion of data and, at the same time, low-cost computation. The adoption of data-intensive ML-algorithms can be found in all application areas of health informatics, and is particularly useful for brain informatics, ranging from basic research to understand intelligence [8] to a wide range of specific brain informatics research [9]. The application of ML methods in biomedicine and health can, for instance, lead to more evidence-based decision-making and helping to go toward *personalized medicine *[10].

According to Tom Mitchell [11], a scientific field is best defined by the questions it studies: ML seeks to answer the question *“How can we build algorithms that automatically improve through experience, and what are the fundamental laws that govern all learning processes?”*

ML is very broad and deals with the problem of extracting features from data to solve predictive tasks, including decision support, forecasting, ranking, classifying (e.g., in cancer diagnosis), detecting anomalies (e.g., virus mutations), or sentiment analysis [12]. The challenge is to discover relevant *structural* patterns and/or *temporal* patterns (“knowledge”) in such data, which are often hidden and not accessible to the human expert. The problem is that a majority of the data sets in the biomedical domain are weakly structured and non-standardized [13], and most data are in dimensions much higher than 3, and despite human experts are excellent in pattern recognition for dimensions $\le 3$, such data make manual analysis often impossible.

Most colleagues from the ML community are concentrating on *automatic* machine learning (aML), with the grand goal of bringing humans-out-of-the-loop, and a best practice real-world example can be found in autonomous vehicles [14].

However, biomedical data sets are full of uncertainty, incompleteness etc. [15], they can contain missing data, noisy data, dirty data, unwanted data, and most of all, some problems in the medical domain are hard, which makes the application of fully automated approaches difficult or even impossible, or at least the quality of results from automatic approaches might be questionable. Moreover, the complexity of sophisticated machine learning algorithms has detained non-experts from the application of such solutions. Consequently, the integration of the knowledge of a domain expert can sometimes be indispensable, and the interaction of a domain expert with the data would greatly enhance the knowledge discovery process pipeline. Hence, *interactive* machine learning (iML) puts the “human-in-the-loop” to enable what neither a human nor a computer could do on their own. This idea is supported by a synergistic combination of methodologies of two areas that offer ideal conditions toward unraveling such problems: human–computer interaction (HCI) and knowledge discovery/data mining (KDD), with the goal of supporting human intelligence with machine intelligence to discover novel, previously unknown insights into data (HCI-KDD approach [16]).

We define iML-approaches as algorithms that can interact withboth computational agents and human agents*) and can optimize their learning behavior through these interactions.

*) In active learning such agents are referred to as the so-called “oracles” [17].

This article is a brief introduction to iML, discussing some challenges and benefits of this approach for health informatics. It starts by motivating the need of a human-in-the-learning-loop and discusses three potential application examples of iML, followed by a very brief overview on the roots of iML in historical sequence: reinforcement learning (1950), preference learning (1987), and active learning (1996). The overview concludes with discussing three examples of potential future research challenges, relevant for solving problems in the health informatics domain: multi-task learning, transfer learning, and multi-agent hybrid systems. The article concludes with emphasizing that successful future research in ML for health informatics, as well as the successful application of ML for solving health informatics problems needs a concerted effort, fostering integrative research between experts ranging from disciplines such as data science to visual analytics. Tackling such complex research undertakings needs both disciplinary excellence and cross-disciplinary networking without boundaries.

## From black-box to glass-box: where is the human-in-the-loop?

The first question we have to answer is: *“What is the difference between the iML-approach to the aML-approach, i.e., unsupervised learning, supervised, or semi-supervised learning?”*

Generally, ML can be categorized into two large subfields: unsupervised learning and supervised learning. The goal in supervised learning (aka predictive learning) is to learn a mapping (prediction) from input data ${\mathbf x}$ to output data *y*, given a (human) labeled set of input-output pairs ${\mathcal {D}}=\{({\mathbf x} _{i},y_i)\}$, where ${\mathcal {D}}$ is the training set containing a number of training samples, e.g., ${\mathbf x _{i}}$ can be a *D*-dimensional vector, called *feature vector*, but it can also be a complex data object (image, graph, time series, etc.). Basically, in supervised learning, the value of the outcome data is based on the number of input data. In unsupervised learning (aka descriptive learning), there are no outcome data, and the goal is to describe the associations and patterns among a set of input data, i.e., we have only given inputs ${{\mathcal {D}}}=\{\mathbf {x}_{i}\}$, and the goal is to discover patterns (aka knowledge) in the data. This is a much more difficult problem.

**Fig. 1:**
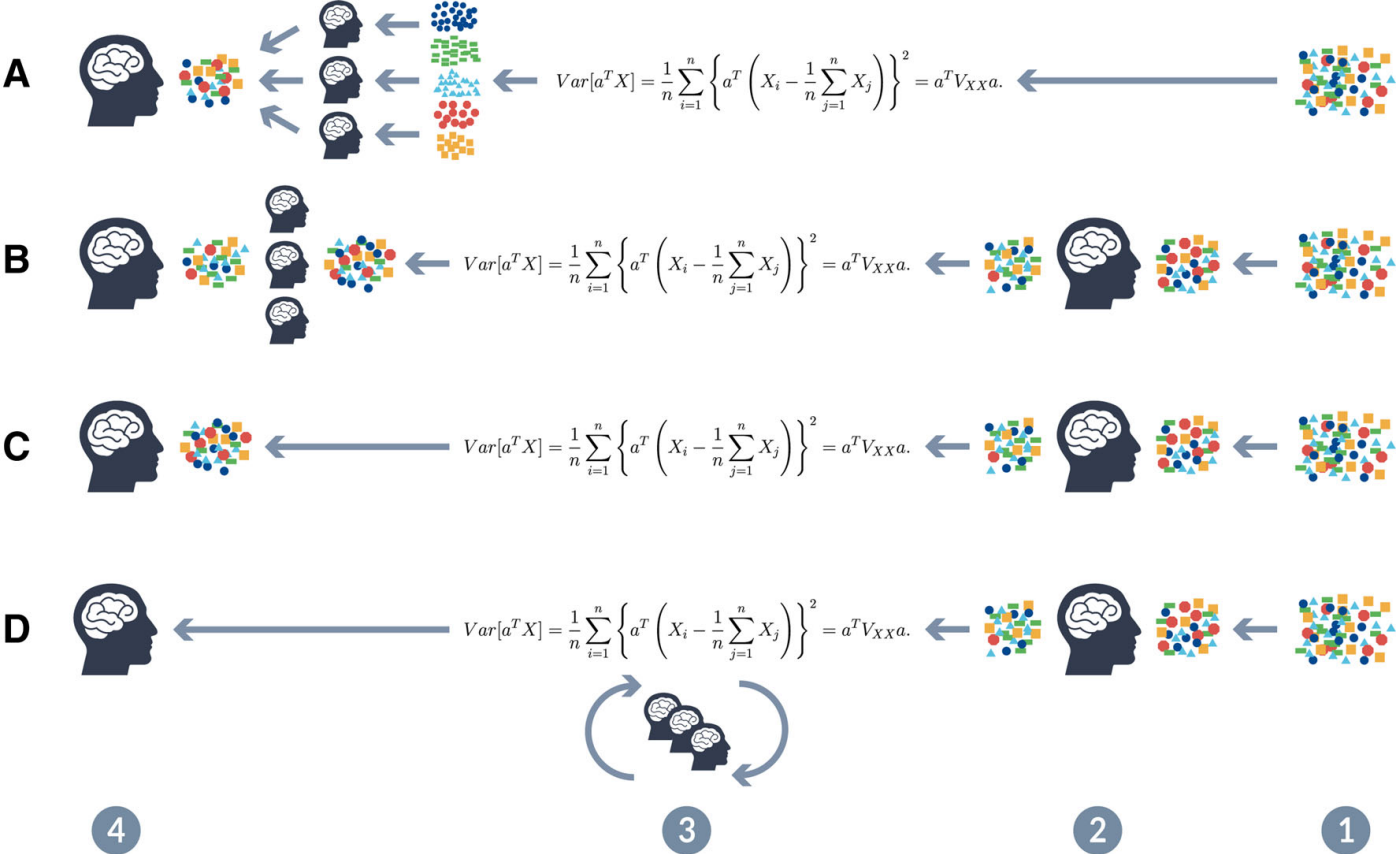
Four different ML-pipelines: *A* unsupervised, *B* supervised—e.g., humans are providing labels for training data sets and/or select features, *C* semi-supervised, *D* shows the iML human-in-the-loop approach: the important issue is that humans are not only involved in pre-processing, by selecting data or features, but actually during the learning phase, directly interacting with the algorithm, thus shifting away the black-box problem to a wished glass-box, *1* input data, *2* pre-processing phase, *3* human agent(s) interacting with the computational agent(s), allowing for crowdsourcing or gamification approaches, *4* final check done by the human expert

Let us visualize the various approaches in Fig. 1:

Scenario A shows typical unsupervised ML: the algorithm is applied on the raw data and learns fully automatic, because it does not require a human to manually label the data. Interestingly, this process is very similar to how humans learn: When a child is learning to see, it is not constantly told what the right answers are, they just watch, and even if its mother is explaining “that is snow”—actually it is very little information for the child. The human brain’s visual system has approx. $10^{14}$ neural connections—but humans live only for $10^9$ seconds. So to learn one bit per second is of not much use, and the only way to achieve $10^5$ bits per second is from the input itself, so the plausible way to deliver such input is massive parallelism, rather than raw speed of the computing elements [3, 18, 19]. However, the human expert can check of course the results at the end of the ML-pipeline (left side Fig. 1A), so even a fully automatic approach requires a kind of human interaction in the form of parameter tuning by the expert. A typical example of unsupervised learning is *clustering* data into groups with uncountable applications and one good example for an unsupervised method is probabilistic latent semantic analysis (PLSA) [20], which is very helpful in text mining [21].

Scenario B is supervised ML, i.e., humans are providing labels for the training data and/or select features to feed the algorithm to learn—the more samples the better—the human expert can check results at the end of the ML-pipeline. A typical example is *classification*, where the goal is to learn a mapping from input data ${\mathbf x }$ to output data *y*, where $y = {1,\ldots ,C}$, with *C* being the number of classes. If $C=2$, this is called binary classification (if $C>2$, this is called multi-class classification). If the class labels are not mutually exclusive (e.g., somebody may be classified as tall and strong), we call it multi-label classification, but this is best viewed as predicting multiple related binary class labels (a so-called multiple output model). When we use the term classification, we will mean multi-class classification with a single output (please refer to [7] for more details).

Scenario C shows semi-supervised ML, a kind of mixture of A and B—mixing labeled and unlabeled data, so that the algorithm can find labels according to a similarity measure to one of the given groups.

Scenario D now shows the iML-approach, where the human expert is seen as an agent directly involved in the actual learning phase, step-by-step influencing measures such as distance, cost functions, etc.

Obvious concerns may emerge immediately and one can argue: what about the robustness of this approach, the subjectivity, the transfer of the (human) agents; many questions remain open and are subject for future research, particularly in evaluation, replicability, robustness, etc.

## Motivation for iML: when is the human-in-the-Loop beneficial?

There is evidence that humans sometimes still outperform ML-algorithms, e.g., in the instinctive, often almost instantaneous interpretation of complex patterns, for example, in diagnostic radiologic imaging: A promising technique to fill the semantic gap is to adopt an expert-in-the-loop approach, to integrate the physicians high-level expert knowledge into the retrieval process by acquiring his/her relevance judgments regarding a set of initial retrieval results [22].

Despite these apparent findings, so far there is little quantitative evidence on effectiveness and efficiency of iML-algorithms. Moreover, there is practically no evidence, *how* such interaction may really optimize these algorithms, even though “natural” intelligent agents are present in large numbers on our world and are studied by cognitive scientists for quite a while [23]. A very recent work is on building probabilistic kernel machines that encapsulate human support and inductive biases, because state-of-the-art ML-algorithms perform badly on a number of extrapolation problems, which otherwise would be very easy to solve for humans [24].

One possible explanation for the dominance of aML-approaches could be that these are much better to evaluate and therefore are more rapidly publishable. In iML-approaches, methodically correct experiments and evaluations are not only much more difficult and time-consuming, but also very difficult or even impossible to replicate, due to the fact that human agents are subjective, individual, and therefore cannot be copied—in contrast to data, algorithms, and computational agents.

However, in biology, biomedicine, and clinical medicine aML-approaches often reach their limits and through “full automation” (press the button and wait) there is often the danger of modeling artifacts (see examples in Sect. 4.1).

In such cases, the inclusion of a “doctor-into-the-loop” [25] can play a significant role in support of solving hard problems (see the examples in the next paragraph), particularly in combination with a large number of human agents (crowdsourcing). From the theory of human problem solving it is known that, for example, medical doctors can often make diagnoses with great reliability—but without being able to explain their rules explicitly. Here, iML could help to equip algorithms with such “instinctive” knowledge and learn thereof. The importance of iML becomes also apparent when the use of automated solutions due to the incompleteness of ontologies is difficult [26].

## Application examples of iML

### Example: subspace clustering

Clustering is a descriptive task to identify homogeneous groups of data objects based on the dimensions (i.e., the values of the attributes). Clustering methods are often subject to other systems, for example, to reduce the possibilities of recommender systems (e.g., Tag-recommender on YouTube videos [27]); clustering of large high-dimensional gene expression data sets has widespread application in -omics [28]. Unfortunately, the underlying structure of these natural data sets is often fuzzy, and the computational identification of data clusters generally requires (human) expert knowledge about cluster number and geometry. The high-dimensionality of data is a huge problem in health informatics in general and in ML in particular, and the curse of dimensionality is a critical factor for clustering: with increasing dimensionality, the volume of the space increases so fast that the available data become sparse, hence, it becomes impossible to find reliable clusters; also the concept of distance becomes less precise as the number of dimensions grows, since the distance between any two points in a given data set converges; moreover, different clusters might be found in different subspaces, so a global filtering of attributes is also not sufficient. Given that large number of attributes, it is likely that some attributes are correlated, therefore clusters might exist in arbitrarily oriented affinity subspaces. Moreover, high-dimensional data likely include *irrelevant* features, which can obscure to find the relevant ones, thus increasing the danger of modeling artifacts (i.e., undesired outcomes or errors which can be misleading or confusing). The problem is that we are confronted with subjective similarity functions; the simplest example is the grouping of cars in a showroom: a technician will most likely group the cars differently than a mother of three kids (cylinder capacity versus storage capacity). This subspace clustering problem is hard, because for the grouping very different characteristics can be used, highly subjective and context-specific ones. What is recognized as comfort for end-users of individual systems can be applied in scientific research for the interactive exploration of high-dimensional data sets [29]. Consequently, iML-approaches can be beneficial to support finding solutions in hard biomedical problems [30]. Actually, humans are quite good in comparison for determining similarities and dissimilarities—described by non-linear multidimensional scaling (MDS) models [31]. MDS models represent similarity relations between entities as a geometric model that consists of a set of points within a metric space. The output of an MDS routine is a geometric model of the data, with each object of the data set represented as a point in an *n*-dimensional space. The similarity between a pair of objects is now taken to be inversely related to the distance between two object points in the space, and the distance between points *i* and *j* can be computed via

$${\mathrm{dissimilarity}}(i,j) = {\left[ {{{\sum \limits _{k = 1}^n {\left| {{X_{ik}} - {X_{jk}}} \right| } }^r}} \right] ^{\frac{1}{r}}},$$

where *n* is the number of dimensions, $X_{ik}$ is the value of dimension *k* for item *i*, and *r* is a parameter that allows different spatial metrics to be used ($r=2$ = standard Euclidian, $r = 1$ city-block metric). For more details refer to [32].

Grouping data sets into clusters based on their similarity is of enormous importance, and the similarity measure is the key aspect of the clustering process. Clustering is usually studied in unsupervised learning settings, but there is a huge problem with real-world data, because such data rarely result from the so-called well-behaved probabilistic models. Consequently, the study of interactive clustering algorithms is a growing area of research: Awasthi et al. [33] studied the problem of designing local algorithms for interactive clustering and proposed an interactive model and provided strong experimental evidence supporting the practical applicability of it. Their model starts with an initial clustering of the data, then the user can directly interact with the algorithm step-wise. In each step, the user provides limited feedback on the current clustering in the form of split-and-merge requests. The algorithm then makes a local edit to the clustering that is consistent with the user feedback. Such edits are aimed at improving the problematic part of the clustering pointed out by the human-in-the-loop. The goal of the algorithm is to quickly converge (using as few requests as possible) to a clustering that the user is happy with, which is called target clustering. More theoretical foundations of clustering with interactive feedback can be found in [34].

### Example: protein folding

In protein structure prediction, there is still much interest in using amino acid interaction preferences to align (thread) a protein sequence to a known structural motif. The protein alignment decision problem (does there exist an alignment (threading) with a score less than or equal to *K*?) is NP-complete, and the related problem of finding the globally optimal protein threading is NP-hard. Therefore, no polynomial time algorithm is possible (unless P = NP). Consequently, the protein folding problem is NP-complete [35]. Health informatics is faced with many problems that (still) require the human-in-the-loop, e.g., genome annotation, image analysis, knowledge-base population, and protein structure. In some cases, humans are needed in vast quantities (e.g., in cancer research), whereas in others, we need just a few very specialized experts in certain fields (e.g., in the case of rare diseases). Crowdsourcing encompasses an emerging collection of approaches for harnessing such distributed human intelligence. Recently, the bioinformatics community has begun to apply crowdsourcing in a variety of contexts, yet few resources are available that describe how these human-powered systems work and how to use them effectively in scientific domains. Generally, there are large-volume micro-tasks and highly difficult mega-tasks [36]. A good example of such an approach is *foldit*, an experimental game which takes advantage of crowdsourcing for *category discovery* of new protein structures [37]. Crowdsourcing and collective intelligence (putting many experts-into-the-loop) would generally offer much potential to foster translational medicine (bridging biomedical sciences and clinical applications) by providing platforms upon which interdisciplinary workforces can communicate and collaborate [38].

### Example: k-anonymization of patient data

Privacy preserving machine learning is an important issue, fostered by anonymization, in which a record is released only if it is indistinguishable from *k* other entities in the data. k-anonymity is highly dependent on spatial locality in order to effectively implement the technique in a statistically robust way, and in high dimensionalities data become sparse, hence, the concept of spatial locality is not easy to define. Consequently, it becomes difficult to anonymize the data without an unacceptably high amount of information loss [39]. Consequently, the problem of k-anonymization is on the one hand NP-hard, on the other hand the quality of the result obtained can be measured at the given factors: *k-anonymity* means that attributes are suppressed or generalized until each row in a database is identical with at least $k-1$ other rows [40, 41]; *l-diversity* as extension of the k-anonymity model reduces the granularity of data representation by generalization and suppression so that any given record maps onto at least *k* other records in the data [42]; *t-closeness* is a refinement of l-diversity by reducing the granularity of a data representation, and treating the values of an attribute distinctly by taking into account the distribution of data values for that attribute [43]; and *delta-presence*, which links the quality of anonymization to the risk posed by inadequate anonymization [44]), but not with regard to the actual security of the data, i.e., the re-identification through an attacker. For this purpose, certain assumptions about the background knowledge of the hypothetical enemy must be made. With regard to the particular demographic and cultural clinical environment this is best done by a human agent. Thus, the problem of (k-)anonymization represents a natural application domain for iML.

###  Interim summary 

Humans are very capable in the explorative learning of patterns from relatively few samples, while classic supervised ML needs large sets of data and long processing time. In the biomedical domain, often large sets of training data are missing, e.g., with rare diseases or with malfunctions of humans or machines. Moreover, in clinical medicine time is a crucial factor—where a medical doctor needs the results quasi in real-time, or at least in a very short time (less than 5 minutes), for example, in emergency medicine or intensive care. Rare diseases are often life threatening and require a rapid intervention—the lack of much data makes aML-approaches nearly impossible. An example for such a rare disease with only few available data sets is CADASIL (Cerebral Autosomal Dominant Arteriopathy with Subcortical Infarcts and Leukoencephalopathy), a disease, which is prevalent in 5 per 100,000 persons and is therefore the most frequent monogenic inherited apoplectic stroke in Germany.

Particularly in the patient admission, human agents have the advantage to perceive the total situation at a glance. This aptitude results from the ability of transfer learning, where knowledge can be transferred from one situation to another situation, in which model parameters, i.e., learned features or contextual knowledge are transferred.

The examples mentioned so far demonstrate that the application of iML-approaches in “real-world” situations sometimes can be advantageous. These examples demonstrate that human experience can help to reduce a search space of exponential possibilities drastically by heuristic selection of samples, thereby helping to solve NP-hard problems efficiently—or at least optimize them acceptably for a human end-user.

## Origins and fundamentals of iML

The basis for iML can be found in three fundamental pillars—in historical sequence: reinforcement learning (RL), preference learning (PL), and active learning (AL), in particular active preference learning (APL).

### Reinforcement learning (RL)

Reinforcement learning (RL) was discussed by Turing [45] and is to date the most studied approach in ML. The theory behind RL is rooted in neuropsychological issues on behavior of how agents may optimize their control of an complex environment. Consequently, RL is a branch of ML concerned with using experience gained through *interacting* with the world and evaluative feedback to improve the ability of a system to generate behavioral decisions. This has been called the *artificial intelligence problem in a microcosm* because learning agents must act autonomously to perform well and to achieve their goals. Driven by the increasing availability of rich data, RL has achieved great results, including developments in fundamental ML-relevant areas, such as generalization, planning, exploration, and empirical methodology, leading to better applicability to real-world problems [46].

RL is different from supervised learning, where learning happens from examples provided by an external (human) supervisor. This is an important kind of learning; however, it alone is not sufficient for learning from interaction. In interactive problems, it is often impractical to obtain examples of desired behavior that are both correct and representative for all the situations in which the agent has to act. In unknown territory (which we want to explore), where one would expect learning to be most beneficial—an agent must be able to learn from its own experience. Despite all limitations, RL is the first field to seriously address the computational issues that arise when learning from interaction with an environment in order to achieve long-term goals, because it makes use of a formal framework defining the interaction between a learning agent and its environment in terms of states, actions, and rewards. This framework is intended to be a simple way of representing essential features of general AI problems and features including a sense of cause and effect, a sense of uncertainty and non-determinism, and the existence of explicit goals [47]. For an overview on some RL-algorithms please refer to [48].

In the typical RL-model, an agent is connected to its environment via perception and action, and in each step of interaction the agent receives as input *i*, some indication of the current state *s*, of the environment, and then the agent chooses an action *a*, which changes the state of the environment, and the value of this state transition is communicated to the agent through a scalar reinforcement signal *r*. The agent’s behavior *B* should now choose actions that tend to increase the long-run sum of values of the RL signal. It can learn to do this over time by systematic trial and error, guided by a wide variety of algorithms. Formally, the model consists of a discrete set of environment states ${\mathcal {S}}$, a discrete set of agent actions ${\mathcal {A}}$, and a set of scalar reinforcement signals, typically [0, 1] or ${\mathbb {R}}$.

An input function *I* determines how the agent views the environment state [49].

A current approach is multi-agent reinforcement learning (MARL), because the complexity of many tasks arising in complex domains makes it difficult to solve them with pre-programmed (single) agent behaviors, instead, agents can discover a solution on their own, using learning. A significant part of the research on multi-agent learning concerns RL-techniques [50].

However, to successfully apply RL in complex real-world situations, agents are confronted with a hard task: they must derive efficient representations of the environment from high-dimensional sensory inputs, and use these to generalize past experience to new situations. It is amazing that humans solve this problem through a harmonious combination of RL and hierarchical sensory processing systems. A big disadvantage is that such approaches have been applied in domains in which useful features can be handcrafted, or in domains with fully observable, low-dimensional state spaces. A very recent awesome work on Atari games demonstrated that a deep Q-network agent, receiving only the pixels and the game score as inputs, was able to surpass the performance of all previous algorithms and achieve a level comparable to that of a professional human games tester, thereby bridging the divide between high-dimensional sensory inputs and actions, resulting in the first artificial agent that is capable of learning to excel at a diverse array of challenging tasks [51], which opens many avenues for further research.

*Interactive RL* is not yet established in the health informatics domain, although there is some previous work from other domains, particularly in training of human-centric robots. The general question is how human interaction can influence the ML process and how natural human feedback can improve standard RL-algorithms [52], [53]. Another example includes a multi-agent-based reinforcement learning algorithm, in which the interactions between travelers and the environment are considered to simulate temporal–spatial characteristics of activity-travel patterns within a town [54]. It remains open for future research to transfer these insights into the health informatics domain.

### Preference learning (PL)

Preference Learning (PL) is aiming to learn a predictive preference model from observations (empirical data) that reveal, explicitly or implicitly, information about the specific *preferences* of a user or a group of users. This can be supported by methods for preference mining, e.g., to gain knowledge about users likes and dislikes to provide personal and custom-tailored recommendations [55]. PL can be seen as a natural link between ML and decision support, and was primarily applied in information retrieval with the central task of *learning to rank* [56, 57]. Characteristic learning tasks include label ranking (see example below), instance ranking, and object ranking. PL is meanwhile an established subfield in ML [58] and a comprehensive and modern overview can be found in [59]. The underlying theoretical basis for PL can be found in *human concept learning,* the ability of humans to think in terms of abstractions: humans are able to order their experience into coherent categories by classifying a new situation as a member of a collection of previous situations for which responses may be appropriate [60].

One of the unsolved problems in the field of (human) concept learning, and which are highly relevant for ML research, concerns the factors that determine the *subjective difficulty of concepts*: why are some concepts psychologically extremely simple and easy to learn, while others seem to be extremely difficult, complex, or even incoherent? These questions have been studied since the 1960s by cognitive scientists, but are still unanswered. Also more recent characterizations of concepts as prototypes rather than logical rules leave it unsolved [61, 62].

The preference learning (PL) model is described by a seminal work of [63]: Let us show to the end-user *M* pairs of items. In each case, the user has to chose which item to be preferred. Consequently, the data set consists of the ranked pairs ${\mathcal {D}} = \{{\mathbf {r}}_{k} \succ {\mathbf {c}}_{k}; k = 1, ..., M\}.$ The symbol $\succ$ indicates the preference of ${\mathbf r _{k}}$ over ${\mathbf c _{k}}$.

The elements ${\mathbf r _{k}}$ and ${\mathbf c _{k}}$ of the ranked pairs ${\mathbf r _{k}} \succ {\mathbf c _{k}}$ are taken from a set of training data with *N* elements.

The goal is to compute the item ${\mathbf x }$ with the highest user valuation in the least comparisons as possible. The valuation functions for ${\mathbf r }$ and ${\mathbf c }$ can be modeled as follows: $u({\mathbf r _{k}}) = f({\mathbf r _{k}}) + e_rk$ and $u({\mathbf c _{k}}) = f({\mathbf c _{k}}) + e_ck.$

In these functions the noise terms are Gaussian, hence, a non-parametric Gaussian process prior to the unknown mean valuation can be assigned [64].

Such random utility models have a long tradition in psychology and economy and go back to Thurstone [65], and Bush and Mosteller [66].

Under the Gaussian utility models, the probability *P* that the item ${\mathbf r}$ is preferred to item ${\mathbf c}$ is given by

$$P({\mathbf {r}}_{k} \succ {\mathbf {c}}_{k}) = P(u({\mathbf {r}}_{k}) > u({\mathbf {c}}_{k})) = \Phi ({\frac{\mathbf {r}_{k}- {\mathbf {c}}_{k}}{\surd 2\sigma }})$$

Fürnkranz et al. [67] integrated both PL and RL: an important motivation for a preference-based approach to reinforcement learning is the observation that in many real-world domains, numerical feedback signals are *not* instantaneously available—as for example in the medical domain—or are defined arbitrarily in order to satisfy the needs of conventional RL-algorithms. The authors proposed an alternative framework for RL, in which qualitative reward signals can be directly used by the learner. Such an approach can be viewed as a generalization of the conventional RL framework in which only a partial order between policies is required, instead of the total order induced by their respective expected long-term reward. The goal was to equip the RL-agent with qualitative “policy models,” such as ranking functions that allow sorting its available actions from the most to the least promising, as well as algorithms for learning such models from qualitative feedback. In an interesting experiment, they applied a model developed by [68] to a simulation of optimal therapy design in cancer treatment. Their framework allows the transfer of the RL approach into a qualitative setting, i.e., the medical domain, where reward is not available on an absolute, numerical scale, instead, the used comparative reward functions for a decision which of two actions is preferable in a given state (label ranking). This is a good example for the human-in-the-loop, where *qualitative feedback* can be used, which cannot be produced by the environment but by a human expert.

Much more work has been done in robot learning, e.g., Knox et al. [69] presented a case study of applying a framework for learning from human feedback to a physically embodied robot. They also provided a demonstration of the ability to train multiple behaviors by such feedback without algorithmic modifications, from free-form human-generated feedback without any further guidance or evaluative feedback. Wilson et al. [70] considered the problem of learning control policies via trajectory preference queries to an expert (trajectory planning is a major challenge in robotics and autonomous vehicles). In particular, the agent confronts a human expert with pairs of policies, and the expert indicates which trajectory is preferred. The agent's goal is to elicit a latent target policy from the human expert with as few queries as possible. To tackle this problem, the authors proposed a Bayesian model of the querying process and introduced two methods that exploit this model to actively select expert queries. Jain et al. [71] considered the problem of learning good trajectories for manipulation tasks, which is challenging because the criteria defining good trajectories varies with users, tasks, and environments. For this purpose, they proposed a co-active online learning framework for teaching robots the preferences of its users for object manipulation tasks. The novelty of their approach was in the type of feedback expected from the user: the human user was not required to demonstrate optimal trajectories as training data, but merely needs to iteratively provide trajectories that slightly improve over the trajectory currently proposed by the system.

In the medical domain—as in many other domains—we have many situations where the creative input of a human is still required, further examples are the fields of active learning, active preference learning, and interactive learning and optimization which we will very briefly discuss next.

### Active learning (AL)

The idea behind *active learning (AL)* is that a ML-algorithm can achieve greater accuracy with fewer training labels, if it is allowed to choose the data from which it learns. An active learner may pose queries, usually in the form of unlabeled data instances to be labeled by an *oracle *(e.g., a human annotator, for example, see [72]). Active learning is well-motivated in many modern machine learning problems, where unlabeled data may be abundant or easily obtained, but labels are difficult, time-consuming, or expensive to obtain [73].

For many types of ML-algorithms, one can compute the statistically *optimal* way to select training data. While the techniques for neural networks are computationally expensive and approximate, the techniques for mixtures of Gaussians and locally weighted regression are both efficient and accurate [74]. A good example was presented by Warmuth et al. [75] wherein they investigated the following data mining problem from computer-aided drug design: From a large collection of compounds, find those that bind to a target molecule in as few iterations of biochemical testing as possible. In each iteration, a comparatively small batch of compounds is screened for binding activity toward this target. They employed AL for selecting the successive batches and the main selection strategy was based on the maximum margin hyperplanes generated by Support Vector Machines. The hyperplane separates the current set of active compounds from the inactive compounds and has the largest possible distance from any labeled compound.

A further approach is interactive Learning and Optimization (ILO), which started with the paper by Brochu et al. [63]—already discussed in the Sect. 5.2. They proposed an AL algorithm to learn a continuous valuation model from discrete preferences. Their algorithm automatically decided what items are best presented to a human in order to find the item that they value highly in as few trials as possible, and exploit so-called quirks; peculiarities of human psychology to minimize time and cognitive burden. For this purpose, their algorithm maximized the expected improvement at each query without accurately modeling the entire valuation surface, which would otherwise be computationally expensive. This problem is hard, due to the fact that the space of choices is infinite. Meanwhile ILO has been applied mostly to information retrieval, for example Yue and Joachims [76] presented an online learning framework, tailored toward real-time learning from observed user behavior in search engines. They only required pairwise comparisons which were shown to be reliably inferred from implicit feedback. In their work, they applied the dueling bandits problem [77]. Progress in this area can lead to cost-effective systems for a variety of application domains such as *personalized search*.

Bayesian approaches to utility elicitation typically adopt (myopic) expected value of information (EVOI) as a natural criterion for selecting queries. However, EVOI-optimization is usually computationally prohibitive.

Viappiani and Boutilier [78] examined the expected value of information (EVOI) optimization using choice queries, i.e., queries in which a user is asked to select his/her most preferred product from a set. They showed that under very general assumptions, the optimal choice query with regard to EVOI coincides with the optimal recommendation set, i.e., a set maximizing the expected utility of the user selection. Since recommendation set optimization is a simpler, sub-modular problem, this can greatly reduce the complexity of both exact and approximate (greedy) computation of optimal choice queries. They also examined the case where user responses to choice queries are error-prone (using both constant and mixed multinomial logit noise models) and provide worst-case guarantees. Finally, they presented a local search technique for query optimization that worked well with large outcome spaces.

An approach can benefit from the ILO scheme in order to gradually shape, for example, the interestingness function (typically not all patterns with same statistical properties are equally interesting) and the methodology (sequences of decisions to process the vast amount of heterogeneous databases) most appropriate to discover such interesting patterns. The question “What is interesting?” is one of the most pressing questions in our fields.

## Future challenges

Much future research has to be done, particularly in the fields of Multi-Task Learning and Transfer Learning to go toward Multi-Agent Hybrid Systems as ultimate applications of the iML-approach.

### Example: multi-task learning

Multi-task learning (MTL) aims to improve the prediction performance by learning a problem together with multiple, different but related other problems through shared parameters or a shared representation. The underlying principle is *bias learning* based on probable approximately correct learning (PAC learning) [79]. To find such a bias is still the hardest problem in any ML task and essential for the initial choice of an appropriate hypothesis space, which must be large enough to contain a solution, and small enough to ensure a good generalization from a small number of data sets. Existing methods of bias generally require the input of a human-expert-in-the-loop in the form of heuristics and domain knowledge to ensure the selection of an appropriate set of features, as such features are the key to learning and understanding. However, such methods are limited by the accuracy and reliability of the experts knowledge (robustness of the human) and also by the extent to which that knowledge can be transferred to new tasks (see next subsection). Baxter [80] introduced a model of bias learning which builds on the PAC learning model which concluded that learning multiple related tasks reduces the sampling burden required for good generalization and that the bias learnt on sufficiently many training tasks is likely to be good for learning novel tasks drawn from the same environment (the problem of transfer learning to new environments is discussed in the next subsection). A practical example is *regularized MTL* [81], which is based on the minimization of regularization functionals similar to Support Vector Machines (SVMs), which have been successfully used in the past for singletask learning. The regularized MTL approach allows to model the relation between tasks in terms of a novel kernel function that uses a taskcoupling parameter and largely outperforms singletask learning using SVMs. However, multi-task SVMs are inherently restricted by the fact that SVMs require each class to be addressed explicitly with its own weight vector. In a multi-task setting this requires the different learning tasks to share the *same set of classes.* An alternative formulation for MTL is an extension of the large margin nearest neighbor algorithm (LMNN) [82]. Instead of relying on separating hyperplanes, its decision function is based on the nearest neighbor rule which inherently extends to many classes and becomes a natural fit for MTL. This approach outperforms state-of-the-art MTL classifiers, however, many open research challenges remain open in this area [83].

### Example: transfer learning (generalization)

A huge problem in ML is the phenomenon of *catastrophic forgetting*, i.e., when having learned one task and being transferred to another task the ML-algorithm “forgets” how to perform the learned task. This is a well-known problem which affects ML-systems and was first described in the context of connectionist networks [84]; natural cognitive systems rarely completely disrupt or erase previously learned information, i.e., natural cognitive systems do not forget “catastrophically” [85]. Consequently, the challenge is to discover how to avoid the problem of catastrophic forgetting, which is a current hot topic [86].

According to Pan and Yang [87], a major assumption in many ML-algorithms is that both the training data and future (unknown) data must be in the same feature space and are required to have the same distribution. In many real-world applications, particularly in the health domain, this is not the case: Sometimes we have a classification task in one domain of interest, but we only have sufficient training data in another domain of interest, where the latter data may be in a completely different feature space or follow a different data distribution.

In such cases transfer learning would greatly improve the performance of learning by avoiding much expensive data-labeling efforts, however, many open questions remain for future research [88].

### Example: multi-agent hybrid systems

Multi-Agent Systems (MAS) are collections of many agents interacting with each other. They can either share a common goal (for example an ant colony, bird flock, or fish swarm), or they can pursue their own interests (for example as in an open-market economy).

MAS can be traditionally characterized by the facts that (a) each agent has incomplete information and/or capabilities for solving a problem, (b) agents are autonomous, so there is no global system control; (c) data are decentralized; and (d) computation is asynchronous [89]. For the health domain, of particular interest is the *consensus problem*, which formed the foundation for distributed computing [90].

The roots are in the study of (human) experts in group consensus problems: Consider a group of humans who must act together as a team and each individual has a subjective probability distribution for the unknown value of some parameter; a model which describes how the group reaches agreement by pooling their individual opinions was described by DeGroot [91] and was used decades later for the aggregation of information with uncertainty obtained from multiple sensors [92] and medical experts [93].

On this basis, Olfati-Saber et al. [94] presented a theoretical framework for analysis of consensus algorithms for networked multi-agent systems with fixed or dynamic topology and directed information flow. In complex real-world problems, e.g., for the epidemiological and ecological analysis of infectious diseases, standard models based on differential equations very rapidly become unmanageable due to too many parameters, and here MAS can also be very helpful [95]. Moreover, collaborative multi-agent reinforcement learning has a lot of research potential for machine learning [96].

Obviously, there would be a lot of opportunities and research challenges in integrating a human agent to form *hybrid systems* [97], and making use of *Multi-Agent Hybrid Systems (MAHS)*: [98].

## Conclusion

There are uncountable future challenges in ML generally and specifically in the application of ML to health informatics. The ultimate goal is to design and develop algorithms which can *automatically *learn from data and thus can improve with experience over time *without any human-in-the-loop.* However, the application of such aML-approaches in the complex health domain seems elusive in the near future and a good example are Gaussian processes, where aML-approaches (e.g., standard kernel machines) struggle on function extrapolation problems which are trivial for human learners. Consequently, iML-approaches, by integrating a human-into-the-loop (e.g., a human kernel [24], thereby making use of human cognitive abilities, seem to be a promising approach. iML-approaches can be of particular interest to solve problems in health informatics, where we are lacking big data sets, deal with complex data and/or rare events, where traditional learning algorithms suffer due to insufficient training samples. Here, the doctor-in-the-loop can help, where human expertise and long-term experience can assist in solving problems which otherwise would remain NP-hard.

Finally, it should be strongly emphasized that successful application of machine learning for health informatics requires a concerted effort of experts from seven different areas including (1) data science, (2) machine learning algorithms, (3) graph theory/network science, (4) computational topology, (5) entropy, (6) data visualization and visual analytics, and (7) privacy, data protection, safety, and security—following the HCI-KDD approach in combining the best of two worlds [16]. For complex research, both disciplinary excellence and cross-disciplinary networking are required.
